# Vaginal microbiota in assisted reproduction: determinants, dynamics, and impact on clinical outcomes

**DOI:** 10.3389/fmicb.2026.1770446

**Published:** 2026-04-10

**Authors:** Xiaoping Liu, Wenting Tang, Congge Li, Yan Jiao, Yun Bai, Lifeng Xiang, Ze Wu

**Affiliations:** 1 Faculty of Life Science and Technology, Kunming University of Science and Technology, Kunming, Yunnan, China; 2 Department of Reproductive Medicine, The First People's Hospital of Yunnan Province, School of Medicine, Kunming University of Science and Technology, Kunming, Yunnan, China; 3 Department of Reproductive Medicine, NHC Key Laboratory of Healthy Birth and Birth Defect Prevention in Western China (Co-building), The First People's Hospital of Yunnan Province, Kunming, Yunnan, China; 4 Department of Reproductive Medicine, The First People's Hospital of Yunnan Province, Kunming University of Science and Technology Affiliated Hospital, Kunming, China; 5 The Affiliated Hospital of Kunming University of Science and Technology, Kunming, Yunnan, China; 6 KUST-YPFPH Reproductive Medicine Joint Research Center, Medical School, Kunming University of Science and Technology, Kunming, Yunnan, China; 7 School of Life Sciences, Yunnan Normal University, Kunming, China

**Keywords:** assisted reproductive technology, *Lactobacillus*, microecological intervention, reproductive outcomes, vaginal microbiota

## Abstract

The vaginal microbiota (VMB), predominantly composed of *Lactobacillus* genus, plays a crucial role in maintaining female reproductive health through acid production, immune modulation, and protection against pathogens. However, substantial inter-individual variability exists in its composition and stability. In assisted reproduction, the vaginal microenvironment is increasingly recognized as an important factor influencing embryo implantation and pregnancy outcomes. Emerging evidence suggests that the composition and dynamics of the vaginal microbiome are not only predictive biomarkers but also potential regulatory targets influencing assisted reproduction outcomes. This review outlines vaginal microbial community types, key behavioral and host-related determinants, and their links to ART outcomes. We also discuss current limitations, including methodological heterogeneity, unclear causal mechanisms, and the lack of standardized intervention strategies. Finally, we highlight the need for longitudinal and multi-omics studies to support the clinical translation of vaginal microbiome research in reproductive medicine.

## 1 Introduction

The vaginal microbiota (VMB), a critical ecological system in the female reproductive tract, maintains mucosal homeostasis and reproductive health through its structural and functional integrity. Characterized by low diversity and *Lactobacillus* dominance in healthy reproductive-aged women, this distinct configuration, first described by Döderlein (1892), is now a established determinant of vaginal health (Galask et al., 1976; Hillier et al., 1993; Sobel, 1999). *Lactobacillus* metabolize glycogen to produce lactic acid, thus maintaining a mildly acidic vaginal environment (pH ≈ 4.0) that inhibits potential pathogens growth (Boskey et al., 1999; O'Hanlon et al., 2013). They also produce antimicrobial factors, including bacteriocins and hydrogen peroxide, contributing to immune regulation and epithelial barrier integrity (Aldunate et al., 2015; Delgado-Diaz et al., 2019).

However, not all women harbor a *Lactobacillus*-dominated VMB. Approximately 25% exhibit a diverse, non-*Lactobacillus*-dominated microbiota enriched with anaerobic species (Zhou et al., 2004; Zhou et al., 2010; Zhou et al., 2007; Verstraelen et al., 2004). Notably, this profile lacks overt symptoms and is often classified as bacterial vaginosis (BV) (Peebles et al., 2019; Allsworth and Peipert, 2007), yet clinical findings may still be considered “normal.” This challenges the traditional dichotomy of infection versus non-infection and supports a continuum model of vaginal microbial states. Increasing evidence links such high-diversity profiles to increased susceptibility to sexually transmitted infections (Gosmann et al., 2017; Brotman et al., 2012) and adverse reproductive outcomes, such as spontaneous preterm birth (Feehily et al., 2020; Elovitz et al., 2019; Freitas et al., 2018; Romero et al., 2014; Kindinger et al., 2016). Elevated microbial diversity is often indicative of impaired vaginal barrier function (Berard et al., 2023), although the underlying molecular mechanisms remain unclear.

With the widespread implementation of assisted reproductive technology (ART), the role of the VMB in fertility treatment has garnered increasing scholarly attention. Evidence indicates that a higher abundance of *Lactobacillus* at embryo transfer is associated with improved implantation and clinical pregnancy rates, whereas reduced *Lactobacillus* and increased microbial diversity are linked to implantation failure and higher miscarriage risk (Haahr et al., 2019; Koedooder et al., 2019; Haahr et al., 2016). During ART procedures, including controlled ovarian stimulation (COS), oocyte retrieval, and embryo transfer, the VMB undergoes dynamic changes influenced by hormonal and clinical factors (Franasiak and Scott, 2015), which may influence pregnancy outcomes. However, the mechanisms underlying these associations remain to be fully elucidated.

This review summarizes the composition, classification, and determinants of the VMB and its clinical relevance in ART. It also highlights current methodological limitations and research gaps, with the aim of supporting the development of standardized analytical approaches and targeted microecological interventions to improve reproductive outcomes.

## 2 Classification framework of the VMB

In healthy women, the VMB is typically dominated by members of the *Lactobacillus* genus. Advances in next-generation sequencing (NGS) have substantially deepened the understanding of VMB profiling, enabling a shift from broad categorization toward more refined community characterization. A landmark study by Ravel et al. (2010), based on 16S rRNA gene sequencing of a multi-ethnic cohort, classified the VMB into five community state types (CSTs) according to microbial composition and relative abundance. Among these, four CSTs are dominated by different *Lactobacillus* species, whereas CST IV represents a heterogeneous, non-*Lactobacillus*-dominated community enriched with anaerobic taxa.

Building upon the foundational CST framework, the Ravel team (France et al., 2020) introduced the VALENCIA algorithm to achieve standardized and higher-resolution community typing. CST I and CST III were subdivided into subtypes A and B according to the relative abundance of their dominant *Lactobacillus*, while CST IV was delineated into seven subtypes characterized by differential predominance of non-Lactobacillus taxa. This approach further stratifies both *Lactobacillus*-dominated and non-*Lactobacillus*-dominated communities based on compositional patterns, thereby improving comparability across studies. In parallel, the VIRGO database (Ma et al., 2020) was developed to enable subspecies-level classification and functional annotation through the integration of genomic and transcriptomic data, providing a platform for more detailed investigation of VMB structure and function.

Further extending this trajectory, the Chen Zijiang team (Qin et al., 2025) applied non-negative matrix factorization (NMF) to a large cohort and proposed an expanded classification system comprising 13 VMB types (Vagitypes). This framework not only offers more granular ecological resolution but also demonstrates remarkable cross-population generalizability, having been validated in cohorts from Canada, Australia, Belgium, South Africa, and South Korea. Consequently, the Vagitype system represents a valuable complementary model for comparative studies and clinical applications in ethnically diverse populations.

Collectively, the progression from the original CST framework to VALENCIA, VIRGO, and the Vagitype system has markedly enhanced the taxonomic resolution, functional annotation, and cross-population applicability of VMB profiling. The Vagitype system further refined community stratification and demonstrated strong generalizability across diverse study cohorts (Table 1). These advances provide a robust foundation for understanding vaginal microbial ecology and its relevance to reproductive health and disease.

**Table 1:** Vaginal microbial community typing (CSTs/Vagitypes).

Typing system	Detailed classification	Key features & clinical relevance
Ravel et al. (2010)	CST I – *L. crispatus* dominant, pH ≈ 4.0 CST II – *L. gasseri* dominant, pH ≈ 5.0 CST III – *L. iners* dominant, pH ≈ 4.4 CST IV – diverse anaerobes (*Gardnerella, Prevotella, Atopobium, Megasphaera, Peptoniphilus, Sneathia* etc.), pH ≈ 5.3 CST V – *L. jensenii dominant*, pH ≈ 4.7	First proposed the five CST framework; Lactobacillus-dominated CSTs associated with low pH and health; CST- IV linked to BV.
France et al. (2020)	CST I-A/I-B – *L. crispatus* dominant (A: higher abundance; B: lower abundance) CST II – *L. gasseri* dominant CST III-A/III-B – *L. iners* dominant (A: higher abundance; B: co-occurs with other taxa) CST V – *L. jensenii* dominant CST IV-A – *Ca. Lachnocurva vaginae* (BVAB1) + Gardnerella CST IV-B – *Gardnerella* + *Atopobium vaginae* CST IV-C0 – balanced, *Prevotella*-rich CST IV-C1 – *Streptococcus* dominant CST IV-C2 – *Enterococcus* dominant CST IV-C3 – *Bifidobacterium* dominant CST IV-C4 – *Staphylococcus* dominant	Established VALENCIA classifier for standardized CST assignment; expanded to 7 major types and 13 subtypes; emphasized CSTs as dynamic states; correlated with Nugent score, pH, ethnicity, and age.
Qin et al. (2025)	13 Vagitypes: I — *L. crispatus*, I-I with *L. vaginalis*, I-II with *L. coleohominis* and *Cutibacterium acnes* II — *L. iners*, II-II with *L. vaginalis* III — *G. vaginalis* IV — *L. gasseri* V — *L. jensenii* VI — *Prevotella amnii* and other Anaerobes VII — *Fannyhessea (Atopobium) vaginae* VIII — *Prevotella bivia, Sneathia, Megasphaera* IX — *Prevotella timonensis, Dialister micraerophilus* X — *Ureaplasma parvum* XI — *Escherichia/Shigella coli* XII — *Enterococcus faecalis, Streptococcus anginosus* XIII — mixed low-abundance taxa (*Gemella, Mobiluncus*, etc.)	Large cohort (6,755 Chinese women); identified 13 Vagitypes with four core species (*L. iners, L. crispatus, G. vaginalis, U. parvum*). Age strongly associated (turning point at 45 years). Some Vagitypes linked to adverse reproductive outcomes.

## 3 Formation and regulatory factors of the VMB

The diversity-based typing of the VMB has revealed its intricate ecological landscape. However, the formation and transition of distinct community types are not stochastic processes but are orchestrated by a dynamic interplay of intrinsic and extrinsic determinants. Host endocrine status, immune responses, and genetic background provide the physiological foundation for community assembly, whereas environmental influences, including lifestyle, sexual behavior, and antibiotic exposure, may perturb its equilibrium. The interplay between these factors underlies the inter-individual variation in the VMB and governs its transitions between eubiotic and dysbiotic states.

### 3.1 Host characteristics shaping the VMB

#### 3.1.1 Genetic background, race, and ethnicity

Pronounced differences in VMB composition have been documented across racial and ethnic groups (Zhou et al., 2007; Serrano et al., 2019). Ravel et al. reported a higher prevalence of CST-IV among Black and Hispanic women compared to White and Asian women (Ravel et al., 2010). Data from the Human Microbiome Project (HMP) further demonstrated that women of European ancestry predominantly exhibit low-diversity, *Lactobacillus*-dominated communities, whereas women of African ancestry frequently harbor high-diversity microbiota dominated by *G. vaginalis* and BV-associated bacteria (BVAB) (Fettweis et al., 2014). Ethnic heterogeneity also extends to the distribution of specific *Lactobacillus* species: *L. iners* is more prevalent among women of African ancestry, while *L. crispatus* predominates among those of European ancestry (France et al., 2020).

Vaginal microbial profiles from Korea, Europe, Canada, and Australia populations display strong similarity to those of the Chinese VaMHP cohort, whereas the South African cohort has more distinct microbial configurations, suggesting that host genetic background plays a critical role in determining microbial community structure (Qin et al., 2025). Twin studies lend further support to this notion: in a Korean cohort, monozygotic twins demonstrated greater microbiota concordance than dizygotic twins, and *Prevotella* abundance was significantly associated with polymorphisms in the IL-5 gene (Si et al., 2017). Similarly, studies in Kenyan women have implicated host genetic variations in innate immunity and cell-signaling pathways as potential modulators of vaginal microbial composition (Mehta et al., 2020). These findings underscore the critical influence of genetic factors. However, the underlying molecular mechanisms remain incompletely elucidated.

#### 3.1.2 Physiological factors

##### 3.1.2.1 Age

Age represents a major determinant of the composition and stability of the VMB. In prepubertal girls, low estrogen levels result in a highly diverse, anaerobe-rich microbiota. During the reproductive period, estrogen-mediated glycogen accumulation fosters the proliferation of Lactobacillus species, leading to the formation of a low-diversity, Lactobacillus-dominated community ecosystem that supports vaginal homeostasis (Cocomazzi et al., 2023; Auriemma et al., 2021). Following menopause, the decline in estrogen concentration reduces *Lactobacillus* abundance, increases microbial diversity, and facilitates the proliferation of potential pathogens. Notably, marked compositional shifts occur after the age of 45, characterized by decreased Lactobacillus abundance, elevated microbial diversity, and the proliferation of potential pathogens—changes that may be further modulated by obstetric history, contraceptive methods, and the frequency of vaginal douching (Qin et al., 2025).

##### 3.1.2.2 Sex hormones

Sex hormones, such as estradiol (E_2_) and anti-Müllerian hormone (AMH), play a central role in regulating microbiota homeostasis. Higher serum concentrations of E_2_ and AMH have been associated with an increased relative abundance of *L. crispatus*, whereas hormonal decline favors the proliferation of facultative anaerobes. Estrogen promotes glycogen synthesis in the vaginal epithelium, providing a metabolic substrate that, after enzymatic degradation by *α*-Amylase into carbohydrates, supports *Lactobacilli* metabolism. The resultant metabolic product, lactic acid, helps maintain a low vaginal pH, thereby exerting potent inhibitory effects on pathogenic bacteria (Boskey et al., 1999; Miller et al., 2016; Spear et al., 2014; Fredricks et al., 2014).

##### 3.1.2.3 Pregnancy and childbirth

Pregnancy and childbirth are also significant determinants of VMB composition. Women with a history of vaginal delivery tend to exhibit higher microbiota diversity, while multiparity is associated with a decrease in *L. crispatus* abundance (Qin et al., 2025) and an increase in *Gardnerella* (Bogado Pascottini et al., 2021; Berry et al., 2021; Kervinen et al., 2022; Romero et al., 2023). Parity functions as a mediating variable between age and microbiota composition, potentially influencing microbial communities through alterations in local anatomical structures and immune microenvironments (Qin et al., 2025).

##### 3.1.2.4 Body mass index (BMI)

Elevated BMI is another important host factor linked to VMB dysbiosis. Overweight/obese women often present with a depletion of lactobacilli, concurrent increases in anaerobes such as *Gardnerella* and *Prevotella*, elevated vaginal pH, and reduced lactic acid production. These changes collectively impair epithelial barrier integrity and heighten susceptibility to BV (Si et al., 2017; Raglan et al., 2021; Brookheart et al., 2019). Obesity may influence the vaginal microenvironment through chronic inflammation, hormonal dysregulation, and metabolic dysfunction (Si et al., 2017; Allen et al., 2021). Animal studies further demonstrate that high-fat diets can reshape the VMB and enhance local γδ T cell-mediated antiviral responses, suggesting that microbe-immune interactions play a regulatory role in obesity-related reproductive tract infections (Park et al., 2022).

##### 3.1.2.5 Psychological stress

Psychological stress, as an exogenous stressor, is notably associated with reductions in lactobacilli abundance, proliferation of anaerobic taxa, increased prevalence of CST-IV communities, and a higher incidence of molecular-BV (Amabebe and Anumba, 2018). Longitudinal analyses indicate that every 5-point increase in perceived stress scale scores corresponds to a 40% rise in the risk of molecular-BV and may impede the restoration of a healthy microbial state (Turpin et al., 2021). Stress likely induces dysbiosis via activation of the HPA axis, elevated cortisol secretion, and local immune suppression, with these effects being particularly pronounced among Black women (Amabebe and Anumba, 2018).

In summary, host-related factors—including genetic background, racial and ethnic variation, and physiological characteristics such as age, hormonal status, pregnancy, metabolic condition, and psychological stress—collectively shape the composition and structure of the VMB. These factors not only account for inter-individual heterogeneity but also influence temporal dynamics across different life stages.

### 3.2 Environmental and behavioral factors

The stability of the VMB is continually modulated by diverse environmental and behavioral factors. Lifestyle, sexual behavior, contraceptive use, hygiene practices, and medical interventions such as antibiotics and probiotics can directly alter the vaginal microenvironment or indirectly influence host immune function, thereby reshaping the composition and functionality of the VMB (Morsli et al., 2024).

#### 3.2.1 Intravaginal practices

IVP, including vaginal douching and other cleansing behaviors, may disrupt the Lactobacillus-dominated ecosystem and increase susceptibility to colonization by anaerobes and Candida, particularly when performed during menstruation (Morsli et al., 2024). Furthermore, socioeconomic status and education level may indirectly affect microbial composition by shaping hygiene awareness and behavioral norms (Dixon et al., 2023).

#### 3.2.2 Sexual behavior

Sexual behavior represents a major exogenous determinant of microbial diversity and stability. A higher lifetime number of female sexual partners (≥4) and having a partner with a history of BV are both significantly associated with an elevated BV risk (Bradshaw et al., 2013). Multiple sexual partners have also been linked to shifts in specific taxa, although overall microbial diversity may remain unchanged (Qi et al., 2025). Penile–vaginal intercourse and sexual activity with non-regular partners can promote colonization by *Lactobacillus iners*, *Gardnerella*, and related lineages, thereby increasing BV susceptibility (Fredricks et al., 2017). Conversely, stable partnerships (>6 months) and regular sexual activity (>1 episode per month) appear to support microbial stability and convergence between partners. Consistently, women engaged in high-risk sexual behavior, such as sex work, exhibit higher microbial diversity and reduced *Lactobacillus* dominance, which may increase susceptibility to sexually transmitted infections and HIV (Mitchell et al., 2017). Similarly, in women who have sex with women (WSW), sexual contact with new partners is associated with increased microbiota diversity, instability, and enrichment of BV-associated bacteria, supporting partner-mediated microbial transmission (Plummer et al., 2019).

#### 3.2.3 Contraceptive methods

Contraceptive methods exert notable regulatory effects on the VMB. Oral contraceptive pills (OCP) facilitate the colonization of hydrogen peroxide-producing *Lactobacillus* species, help maintain CST-I dominance, and reduce the risk of BV (Morsli et al., 2024). Injectable contraceptives similarly support Lactobacillus predominance but are often accompanied by increased abundance of *Atopobium vaginae* or *Prevotella bivia*. In contrast, the levonorgestrel-releasing intrauterine system (LNG-IUS) is associated with reduced *Lactobacillus* abundance and CST instability, while copper intrauterine devices (Cu-IUD) more frequently result in non-Lactobacillus-dominated communities and vaginal inflammation (Achilles et al., 2018). Condom use was associated with lower Shannon diversity and higher abundance of *A. vaginae* and *P. bivia* (Qi et al., 2025).

#### 3.2.4 Smoking and alcohol

Smoking and alcohol consumption are both recognized risk factors for VMB dysbiosis. Smoking demonstrates a dose–response relationship with BV incidence, with a threshold effect observed at ≥30 cigarettes per week (Bradshaw et al., 2013). The underlying mechanisms may involve biogenic amine accumulation, altered vaginal pH, and immune suppression (Morsli et al., 2024). Alcohol consumption may similarly promote microbial imbalance through comparable pathways and often interacts synergistically with other factors such as BMI and sexual behavior (Morsli et al., 2024; Dixon et al., 2023).

External factors influence the VMB by altering the local environment or modulating host immunity. Diet, sexual behavior, contraceptive use, hygiene practices, and antibiotic exposure can either maintain *Lactobacillus*-dominated communities or promote dysbiosis characterized by increased diversity and anaerobic predominance. However, variable responses to similar exposures suggest that these effects interact with host factors and microbial traits to jointly regulate VMB composition.

### 3.3 Microbiota-associated factors

The stability of the vaginal microecology relies on both the internal dynamics within the microbial community and its ability to resist exogenous microbial invasion. Under healthy conditions, *Lactobacillus* species maintain an acidic environment through the production of lactic acid, hydrogen peroxide, and bacteriocins, thereby suppressing pathogen proliferation and preserving mucosal barrier integrity (Kwon and Lee, 2022). When *Lactobacillus* dominance declines, dysbiosis often manifests as a transition toward CST-IV communities enriched in *G. vaginalis*, *A. vaginae*, *Prevotella* spp., *Fannyhessea vaginae*, *Mobiluncus* spp., and BVAB. These organisms can degrade lactic acid, elevate vaginal pH, and secrete proteases and adhesins that compromise the epithelial barrier, triggering inflammatory cascades and increasing susceptibility to BV, pelvic inflammatory disease (PID), and sexually transmitted infections such as HIV and HPV (Bradshaw et al., 2025; d'Enfert et al., 2020; MacAlpine and Lionakis, 2024).

Moreover, certain bacteria of oral or intestinal origin (such as *Shuttleworthia*, *Bifidobacterium*, and *Streptococcus*) may ectopically colonize the vaginal tract, disrupting microbial homeostasis, particularly after practices such as oral sex or medical instrumentation. This process is facilitated by sialic acid cleavage activity within the VMB, which enables the colonization and persistence of oral-derived *Fusobacterium nucleatum* in sialidase-positive vaginal environments (Cadwell et al., 2020). Ecological competition also exists among *Lactobacillus* species. *L. crispatus* is typically associated with a healthy, stable microbiome, whereas *L. iners* often predominates during transitional states or at the onset of BV, exhibiting lower ecological stability and potential associations with mucosal inflammation (Zhu et al., 2024).

**Figure 1:**
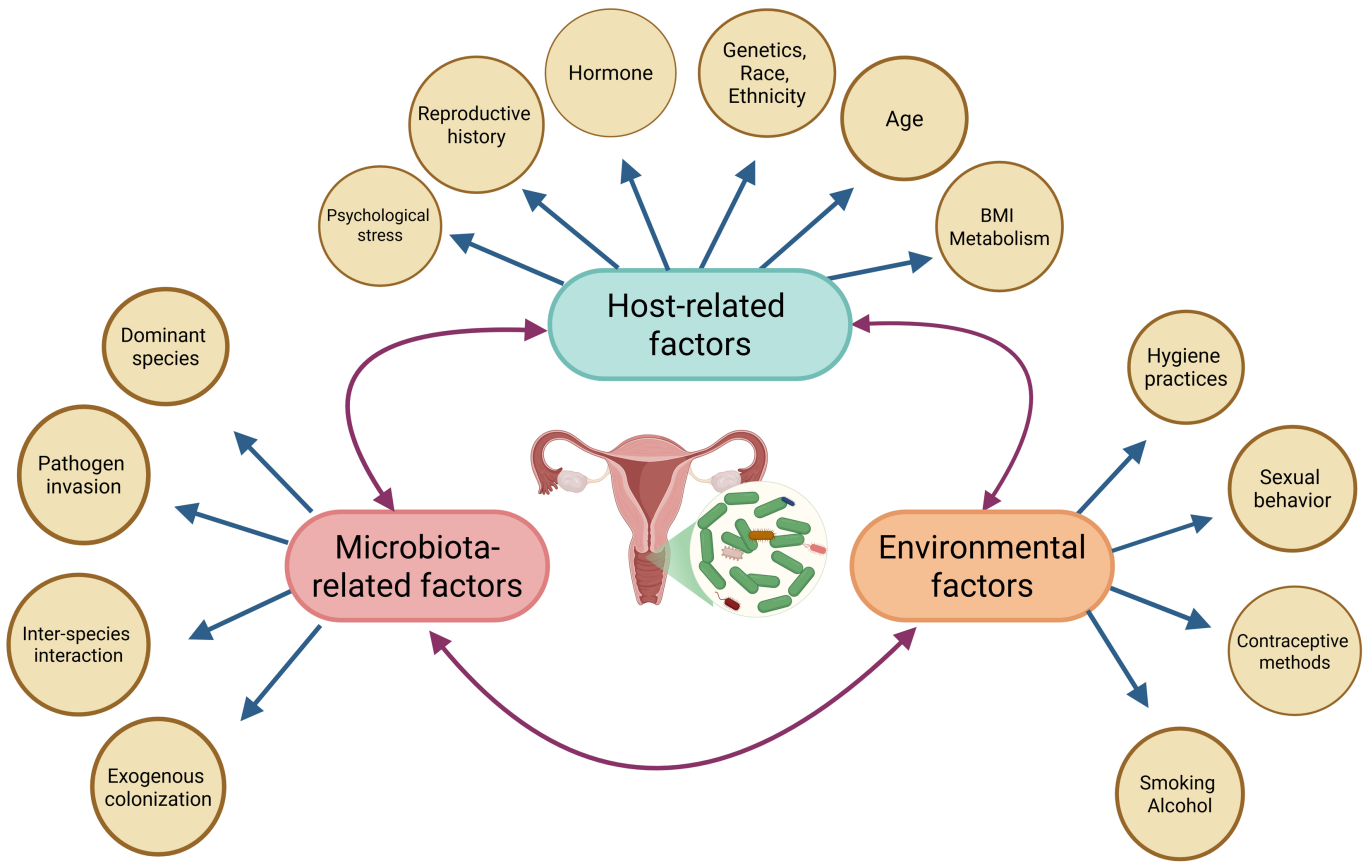
Factors influencing the VMB. The VMB is regulated by the interplay of host-related, environmental, and microbiota-related factors. Host factors, environmental influence, and microbial dynamics collectively shape vaginal microbial balance and reproductive health. (created in Biorender.com by Xiaoping Liu).

The formation and regulation of the VMB result from multi-factorial interactions. Host genetics, ethnicity, and physiological status provide the foundation for microbial colonization, while external factors—such as lifestyle, sexual behavior, and medical interventions—shape its dynamic composition. Interactions within the microbial community further determine its stability and function. Together, these factors contribute to the diversity and heterogeneity of vaginal microecology among individuals (Figure 1).

## 4 Impact of VMB during ART treatment

During COS in IVF/ICSI-ET cycles, patients are exposed to supraphysiological levels of E₂ and progesterone (P) (Pereira et al., 2017; Parisi et al., 2023), which induce significant fluctuations in vaginal microbial composition, relative abundance of Lactobacillus, and CSTs (Zhao et al., 2020; Tian et al., 2024; Carosso et al., 2020; van den Tweel et al., 2024). These hormonal interventions are frequently associated with a decline in Lactobacillus abundance and an increased incidence of BV. Specifically, COS and hormonal exposure often drive a shift from healthy *L. crispatus*-dominated communities toward *L. iners*-dominated or non-Lactobacillus profiles. Such dysbiotic transitions may adversely affect embryo implantation and pregnancy outcomes. In certain patients, the VMB may deteriorate during treatment (van den Tweel et al., 2024). These observations underscore the high responsiveness of the VMB to ART interventions, highlighting the need for dynamic monitoring and tailored interventional strategies (Table 2).

**Table 2:** Summary of Studies Investigating the Effects of COS on VMB in ART Patients.

Author/year	Population	COS protocol	Sample size	Sampling site	Sampling time	Detection method	Main findings
Chen et al. (2025a)	Patients undergoing fresh embryo transfer/antagonist protocol/hormone replacement cycle for frozen embryo transfer	/	92 women	Upper vagina and posterior fornix	Before embryo transfer	16S rRNA sequencing	Elevated estradiol levels increased VMB α-diversity;β-diversity was altered; CSTs shifted toward a *Lactobacillus*- dominant profile.
Yang et al. (2024)	Patients undergoing frozen single blastocyst transfer	/	20 women	Vaginal anterior fornix secretions	Menstrual cycle days 2_5, day 7 of estrogen replacement therapy, and embryo transfer day during pregnancy	16S rRNA sequencing; targeted metabolomics	Significant differences were observed in vaginal microbial composition and metabolic profiles across different time points.
van den Tweel et al. (2024)	Patients undergoing mild ovarian stimulation with IUI or IVF/ICSI	/	53 women (140 samples)	Vaginal posterior fornix	At first visit, before IUI/oocyte retrieval, and before embryo transfer in FET cycle	16S rRNA sequencing qPCR	ART_treatment showed reduced vaginal *Lactobacillus* abundance; CST composition changed significantly in some individuals.
Tian et al. (2024)	Patients undergoing IVF/ICSI with fresh embryo transfer	Long follicular phase protocol	440 women	Vaginal posterior fornix and upper vagina	Within 30 days before downregulation and on hCG trigger day	Routine vaginal smear	Compared with pre_COS, *Lactobacillus* abundance decreased after COS; 16.6% of patients exhibited dysbiotic microbiota;incidence of BV and unexplained dysbiosis significantly increased after COS.
Zhao et al. (2020)	Patients with secondary infertility undergoing IVF	Agonist protocol	122 women (30 with secondary infertility, 40 healthy women in ovulatory phase, 52 healthy women in follicular phase)	Vaginal posterior fornix	Follicular phase day 3, ovulatory phase day 3, after COS + hCG	16S rRNA sequencing	COS + hCG had no significant effect on vaginal microbial abundance in infertile women.
Carosso et al. (2020)	IVF- treatment	Antagonist protocol	15 women	Vaginal posterior fornix	Luteal phase before COS and fresh embryo transfer day	16S rRNA sequencing	After COS + progesterone treatment, Lactobacillus abundance decreasedwhile potentially pathogenic genera increased; overall vaginal microbial diversity significantly increased.

During COS, patients commonly experience increased vaginal discharge and vulvar discomfort. Administration of high-dose ovulation induction agents within a short time frame results in a rapid rise in estrogen levels that exceeds the physiological range, causing decreased vaginal pH and disruption of vaginal microecology. While one study reported that supraphysiological E₂ levels may transiently favor a *Lactobacillus*-dominated microbiome, this shift did not correlate with improved reproductive outcomes (Chen et al., 2025a). *Streptococcus* species were identified as potential microbial effectors mediating the influence of E₂ on pregnancy success. These findings suggest that ART protocols should not only aim to optimize follicular development but also consider the modulatory effects of elevated E₂ on the VMB. Moreover, microbial markers such as *Streptococcus* may hold promise as novel predictors of ART outcomes.

In hormone replacement therapy–frozen embryo transfer (HRT–FET) cycles, *α*-diversity of the vaginal and cervical microbiota is highest during the early menstrual phase. Studies have identified significant differences in specific microorganisms (such as *Peptoniphilus* and *Klebsiella*) and metabolites (including 3-hydroxybenzoic acid, isocitric acid, and quercetin) between pregnant and non-pregnant groups at various time points throughout the cycle—after estrogen administration, on the day of endometrial transformation, and on the day of embryo transfer (Yang et al., 2024). Notably, correlations were identified between certain microbial taxa (particularly *Klebsiella*) and specific metabolites. The dynamic changes in microbial and metabolic profiles may serve as valuable biomarkers for predicting pregnancy outcomes in frozen embryo transfer cycles.

## 5 VMB and reproductive health

### 5.1 Impact on natural fertility

Women affected by infertility exhibit distinct alterations in their VMB compared to healthy reproductive-aged women, primarily characterized by a reduction in Lactobacillus-dominated communities and an increase in microbial diversity (Chen et al., 2025b). Specifically, there is a decline in beneficial species such as *L. crispatus*, *L. gasseri*, and *L. jensenii*, alongside a relative predominance of *L. iners*—a species associated with low ecological stability and limited protective capacity. Concurrently, the prevalence of anaerobic and opportunistic pathogenic bacteria rises, reflected in significantly higher detection rates of BV and CST-IV profiles. Some studies further suggest that lower species diversity, coupled with elevated abundances of *L. iners*, *L. gasseri*, and *G. vaginalis*, may contribute to reduced fertility potential in women (Hasan et al., 2024).

This state of microbial dysbiosis is often accompanied by elevated concentrations of local proinflammatory cytokines (e.g., IL-1β, TNF-*α*) and reduced levels of anti-inflammatory mediators, which may impair conception by disrupting the uterine environment, compromising endometrial receptivity, and facilitating ascending infections (Gao et al., 2024). Moreover, specific bacterial species such as *Fannyhessea vaginae* and *Mobiluncus* spp. have been closely linked to infertility-related pathologies, including tubal obstruction, cervicitis, and PID, underscoring the crucial role of VMB imbalance in the pathogenesis of infertility (Salliss et al., 2022).

Disruption of the vaginal microecology may impair natural fertility through multiple pathways. Persistent BV has been associated with a 43% reduction in fecundity compared to maintaining optimal vaginal health across menstrual cycles (Lokken et al., 2021). A systematic review reported a BV prevalence of 19% among infertile women, significantly higher than that observed in women with normal fertility (van Oostrum et al., 2013). *Lactobacillus*, *Gardnerella*, and *Mobiluncus* have been identified as key microbial predictors of fertility status (Hong et al., 2025).

During follicular development, Gram-negative bacteria associated with BV produce lipopolysaccharide (LPS), which can trigger immune activation even at low concentrations. This initiates a chronic low-grade inflammatory response and promotes the accumulation of reactive oxygen species (ROS), ultimately compromising oocyte quality and leading to anovulation or ovulatory dysfunction (Boots and Jungheim, 2015). Notably, the incidence of BV in women with ovulatory disorder-related infertility ranges from 33 to 36%, compared with 12–15% among women with infertility due to other etiologies (Wilson et al., 2002), suggesting that BV may impair fertility by interfering with ovulatory processes.

BV is also strongly associated with tubal factor infertility. A meta-analysis demonstrated that women with BV have a 2.77-fold higher risk of tubal infertility compared to those with normal vaginal flora (van Oostrum et al., 2013). The underlying mechanism may involve recurrent BV episodes that predispose individuals to subclinical PID. Although many affected women lack a documented history of PID, clinical testing often reveals evidence of past infection with *Neisseria gonorrhoeae* or *Chlamydia trachomatis*. Studies have shown that BV-positive women exhibit markedly elevated infection rates with these pathogens (13% for *Chlamydia*, 26% for *Neisseria*), which are established causes of hydrosalpinx, pelvic adhesions, and tubo-ovarian abscesses. Wang Jinfeng et al. proposed that BV may compromise the endometrial environment through ascending infection (Wang et al., 2021). However, evidence remains limited regarding whether BV alters endometrial receptivity during the implantation window, thereby impairing natural conception.

### 5.2 Impact on ART outcomes

The VMB exerts a profound influence on ART outcomes. Emerging evidence indicates that both microbial composition and inflammatory status collectively affect fertility, serving as potential predictive tools in reproductive medicine (Bar et al., 2025). Women with dysbiotic VMB undergoing IVF-ET exhibit a markedly elevated risk of pregnancy failure (Ji et al., 2022; Miyagi et al., 2022). A systematic review and meta-analysis encompassing 1,095 women undergoing ART reported a BV prevalence of 18.4%, with BV significantly associated with impaired early embryonic development (Singer et al., 2019), highlighting its potential to compromise embryo implantation and early pregnancy maintenance.

Pre-transfer VMB composition has been demonstrated to influence ART outcomes in women with polycystic ovary syndrome and tubal factor infertility (Zhao et al., 2025). Dysbiosis may disrupt the endometrial immune microenvironment through ascending colonization, resulting in elevated local pro-inflammatory cytokines and diminished endometrial receptivity, ultimately reducing clinical pregnancy rates (Haahr et al., 2016). This hypothesis is supported by multiple studies (Wang et al., 2021; Thanaboonyawat et al., 2023; Jones et al., 2019).

Furthermore, BV has been linked to an increased risk of miscarriage following ART treatment. In a cohort of 759 pregnant women, BV-positive individuals experienced significantly higher rates of pregnancy loss before 25 weeks of gestation (Donders et al., 2009). Ascending infection by BV-associated bacteria may induce chorioamnionitis or preterm premature rupture of membranes, contributing to adverse pregnancy outcomes (Guo et al., 2024). Women with recurrent implantation failure (RIF) display evidently reduced detection of vaginal lactobacilli compared to controls, suggesting that impaired lactobacillus-dominated communities are linked to implantation failure (IF) and may serve as a biomarker for RIF (Ichiyama et al., 2021).

Imbalances in the VMB and associated metabolic activities represent critical determinants of embryo implantation. Abnormal or high-diversity microbial profiles are negatively associated with clinical pregnancy rates (Haahr et al., 2019; Haahr et al., 2016), whereas a moderate *Lactobacillus* abundance (~80%) and CST I are linked to improved pregnancy and live birth outcomes (Koedooder et al., 2019; Wang et al., 2024; Karaer et al., 2021). Metagenomic functional analyses indicate enrichment of the L-lysine biosynthesis pathway in women with IF, alongside elevated vaginal pH and reduced Lactobacillus abundance. Moreover, the frequency of failed implantation attempts correlates with the degree of genital tract microbiota disruption, and microbial community profiling can effectively discriminate between patients experiencing repeated IF and those achieving successful implantation (Su et al., 2024). These findings suggest that targeting microbiota-associated metabolic pathways may improve ART outcomes and provide potential biomarkers for clinical prediction and intervention (Zhang et al., 2025).

In summary, maintaining a balanced vaginal microecological environment is essential for both natural conception and assisted reproductive success. Dysbiotic states, including BV, can compromise fertility through multiple pathways—disrupting folliculogenesis, ovulation, tubal function, endometrial receptivity, and early embryonic development—thereby constituting a significant risk factor for impaired reproductive outcomes (Table 3).

**Table 3:** Summary of clinical studies on the association between VMB and pregnancy outcomes in ART patients.

Author/year	Population	Sample size	Sampling site	Sampling time	Detection method	Outcome measure	Main findings
Qin et al. (2025)	IVF treatment fresh embryo transfer	845 cases	Posterior vaginal fornix	/	16S rRNA sequencing	Clinical pregnancy rate, live birth rate	Vagitype dominated by *L. crispatus, L. iners*, and *L. jensenii* showed higher clinical pregnancy and live birth rates, while F. vaginae-dominant Vagitype VII showed the lowest rates.
Chen et al. (2025a)	IVF treatment	294 cases	Posterior vaginal fornix	/	16S rRNA sequencing	Clinical pregnancy	Women with *Lactobacillus*-dominant VMB had higher pregnancy rate, lower microbial diversity, and lower local inflammation; changes in dominant bacteria may affect pregnancy outcomes.
Zhao et al. (2025)	IVF treatment	122 cases	Anterior vaginal wall (upper 1/3) posterior fornix	24 h before ET/LH + 6–8	16S rRNA sequencing	Clinical pregnancy	The infertility group had higher vaginal microbial diversity than the pregnancy group, which was dominated by *Lactobacillus*; high diversity before ET was significantly associated with implantation failure.
Wang et al. (2024)	IVF treatment	1,411 cases	Posterior vaginal fornix	Menstrual phase (days 15–18), secretory phase, implantation phase	16S rRNA sequencing; bacterial culture	Clinical pregnancy rate	*Lactobacillus* abandance was significantly higher in the pregnancy group; vaginal microecology was closely associated with embryo implantation rate.
Su et al. (2024)	Infertility	256 cases	Lower 1/3 vagina, posterior fornix	LH + 7 and embryo transfer day	16S rRNA sequencing	Clinical pregnancy	The pregnancy group had a higher abundance of *Lactobacillus*-dominant microbiota; a post-ET decrease in *Lactobacillus* abundance was associated with reduced pregnancy rates.
Wei et al. (2024)	Frozen embryo transfer	379 cases	Upper 1/3 of lateral vaginal wall	Embryo transfer day	16S rRNA sequencing; vaginal microecology test	Clinical pregnancy	*Lactobacillus* abundance was significantly higher in the pregnancy group; lower microbial diversity was associated with better pregnancy outcomes.
Miyagi et al. (2022)	Frozen embryo transfer	35 cases	Vaginal swab	Days 8–10 of menstrual cycle	16S rRNA sequencing	Clinical pregnancy	*Lactobacillus* abundance was significantly higher in pregnant women than in non-pregnant women.
Ji et al. (2022)	Frozen embryo transfer	275 cases	Upper and middle vagina	Endometrial window (late follicular, luteal, and transfer day)	16S rRNA sequencing; Nugent score	Clinical pregnancy	*Lactobacillus* abundance was significantly higher in the pregnancy group; dysbiosis was associated with lower pregnancy rates.
Karaer et al. (2021)	ART- treatment	223 cases	Posterior vaginal fornix	Before fresh embryo transfer	16S rRNA sequencing	Clinical pregnancy rate implantation rate	Patients with CST I (*L. crispatus*-dominant) had significantly higher pregnancy and implantation rates than non–CST I groups.
Diaz-Martínez et al. (2021)	Frozen embryo transfer	48 cases	Posterior vaginal fornix	FET cycle (secretory phase, transfer day, confirmation of pregnancy)	16S rRNA sequencing	Clinical pregnancy	Pregnant women had higher *Lactobacillus* abundance and lower microbial diversity.
Koedooder et al. (2019)	IVF/ICSI- treatment	303 cases	Vaginal pH 3–5 cm	Before entering IVF/ICSI cycle	16S–23S rRNA sequencing	Clinical pregnancy	Women with higher vaginal *Lactobacillus* abundance had higher pregnancy rates.
Haahr et al. (2019)	IVF- treatment	120 cases	Posterior vaginal fornix	First visit	16S rRNA sequencing, qPCR	Clinical pregnancy rate live birth rate	Women with higher vaginal microbial diversity had lower pregnancy and live birth rates.
Haahr et al. (2016)	Infertility	130 cases	Posterior vaginal fornix	Within 2 months before embryo transfer	PCR	Clinical pregnancy	qPCR analysis showed that higher vaginal microbial diversity was associated with lower pregnancy rates.

## 6 Intervention strategies: microbial applications in ART

Homeostasis of the VMB plays a pivotal role in female fertility and ART outcomes. The dominant genus, Lactobacillus, contributes to reproductive tract health through multiple mechanisms, including competitive epithelial colonization, glycogen metabolism leading to lactic acid production and pH reduction, secretion of antimicrobial substances (e.g., bacteriocins and H₂O₂), and modulation of local immune responses. Dysbiosis, characterized by reduced Lactobacillus abundance and increased anaerobic bacteria, is strongly associated with infertility and impairs ART success, establishing it as a critical target for clinical intervention (Valenti et al., 2018; Kovachev, 2018).

Probiotic therapy represents a cornerstone of microbial modulation in ART. Vaginal administration of live Lactobacillus preparations has demonstrated significant efficacy. However, outcomes are highly dependent on the dosing regimen. In HRT-FET cycles, initiating a 6-day course at the onset of luteal support has been linked to increased live birth rates and reduced miscarriage risk (Thanaboonyawat et al., 2023), whereas single-dose administration post-oocyte retrieval failed to produce significant improvements (Gilboa et al., 2005). Oral supplementation with specific strains, such as *Lactobacillus crispatus* M247, may benefit specific subgroups, including women of advanced reproductive age (30–40 years), those with higher BMI (>22), or those undergoing blastocyst transfer, although further validation is required (Di Pierro et al., 2023).

Lactoferrin, a broad-spectrum antimicrobial and immunomodulatory agent, can be administered vaginally to emulate the protective effects of a healthy microbiota in dysbiotic conditions, complementing probiotic strategies (Valenti et al., 2018). Evidence also indicates that vaginal Lactobacillus supplementation can improve clinical pregnancy rates without increasing miscarriage risk (Wei et al., 2024). Microbiota transplantation represents a promising, though still exploratory, therapeutic approach. Fecal microbiota transplantation (FMT) aims to indirectly modulate hormonal balance and vaginal inflammation—such as Gardnerella overgrowth—by correcting intestinal dysbiosis (e.g., Proteobacteria-driven LPS-induced inflammation) (Quaranta et al., 2019; Yu et al., 2023). In contrast, vaginal microbiota transplantation (VMT) directly restores healthy microbial communities, re-establishing Lactobacillus dominance and associated functions (e.g., H₂O₂ production, pH reduction) (Tuniyazi and Zhang, 2023). However, the long-term safety and efficacy of both approaches remain under investigation.

Integrative analyses of vaginal and gut microbiomes, coupled with host molecular markers such as miRNAs, have facilitated the development of personalized dietary, probiotic, and nutraceutical interventions for patients with repeated ART failure. Implementation of these tailored regimens over 60–90 days has demonstrated marked improvements in reproductive outcomes (Meng et al., 2024). In a cohort of 287 patients with recurrent ART failure, biochemical pregnancy rates reached 75% and clinical pregnancy rates 54.7%.

Maintaining VMB balance is fundamental to reproductive health, and its disruption represents a significant risk factor for impaired ART outcomes. Among current intervention strategies, vaginal probiotic administration and personalized nutritional regulation have demonstrated tangible benefits, whereas lactoferrin may serve as an adjunct therapy. Microbiota transplantation, while promising, requires additional evidence to confirm safety and efficacy. Future efforts should focus on optimizing probiotic strain selection, timing of administration, and synergistic modulation of the gut–vaginal axis, with the ultimate goal of developing precision microbial ecological strategies to improve ART success rates.

## 7 Research limitations and future directions

**Figure 2:**
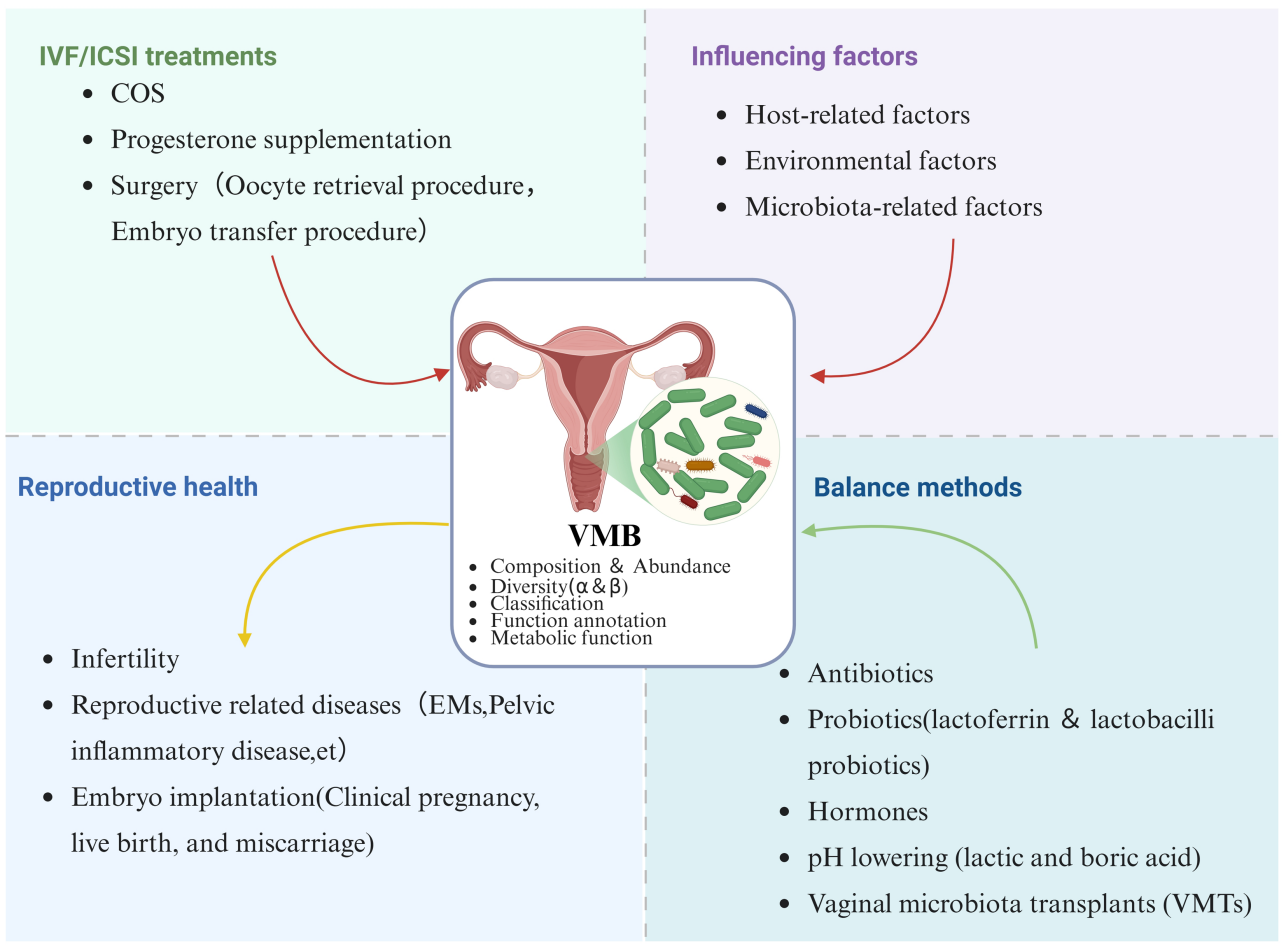
Overview of the VMB and its relevance to reproductive health. The VMB is influenced by host-related, environmental, and microbiota-related factors, as well as by clinical interventions such as IVF/ICSI treatments (e.g., COS, progesterone supplementation, and surgical procedures). VMB composition, diversity, and metabolic function are closely associated with reproductive outcomes, including infertility, reproductive tract diseases, and embryo implantation success. Balance of the VMB can be maintained or restored through antibiotics, probiotics, hormonal regulation, pH-lowering agents, and VMT (created in Biorender.com by Xiaoping Liu).

The VMB is a pivotal determinant of female reproductive health. A healthy vaginal microenvironment is typically dominated by Lactobacillus species, which maintain a low pH, preserve epithelial barrier integrity, and modulate local immune responses. Nevertheless, VMB composition and stability are influenced by a complex interplay of host-related factors, such as age, hormonal status, BMI, and genetic background, as well as lifestyle variables (including sexual activity, hygiene practices, and contraceptive use). Dysbiosis, particularly BV, has been consistently associated with natural infertility, tubal factor infertility, impaired embryo implantation, and adverse outcomes in ART. Furthermore, ART procedures themselves, including hormonal interventions and clinical procedures, may disturb the VMB, increasing the risk of pregnancy loss and miscarriage (Figure 2).

Despite accumulating evidence linking VMB composition to reproductive outcomes, the underlying mechanisms remain incompletely understood, and findings across studies are often inconsistent. Empirical support for microbiota-targeted interventions is still limited. Several critical questions remain unresolved, including how the VMB influences endometrial receptivity, whether it can serve as a reliable predictive biomarker of pregnancy, and the clinical efficacy of microbiome-based interventions (e.g., probiotics and prebiotics) in ART. In addition, methodological heterogeneity—including differences in VMB classification, sampling timing during ART cycles, and definitions of pregnancy outcomes—further complicates cross-study comparisons and clinical translation.

Future research should prioritize longitudinal, multi-timepoint monitoring of the VMB across reproductive and ART stages, combined with mechanistic studies to clarify causal pathways. Developing predictive models and targeted interventions based on microbial profiles may enable personalized, microbiota-informed strategies in reproductive medicine, ultimately improving ART outcomes.
